# A Serious Drug Fraud Detected

**Published:** 1917-05

**Authors:** 


### Abstracts and Selections.

### A Serious Drug Fraud Detected.

As a result of an investigation made by inspectors of the Bureau of Foods and Drugs of the Department of Health it was found that many dealers were handling a spurious product purporting to be Neosalvarsan (.9 gram size). Several arrests have been made for violation of the Sanitary Code in this connection, and the following letter has been prepared for distribution to the drug trade and is herewith reproduced for the information of our readers:

"As a result of an investigation conducted by the Bureau of Food and Drugs, it has been found that there is on the market a spurious drug proporting to be 'Neosalvarsan .9.' This spurious product has no medicinal value (samples analyzed proved to be nothing but common salt and a dirty yellow coloring matter), and it is one of the most despicable frauds ever perpetrated on the public. In order to eliminate this spurious product from the market, the department must have the whole-hearted support of dealers in drugs in the city of New York.

"The fraudulent preparation has been made to imitate the genuine product to such an extent that it requires careful examination by experts to determine whether or not it is the genuine product. The labels, aluminum container, the impressed trade mark on the cap of the aluminum container, the advertising matter inside the package, and the glass ampoule, all have been prepared so as to deceive and mislead, not only the public, but drug dealers and physicians as well.

"Under the circumstances, the Department of Health wishes to call your attention to the matter and requests that you immediately communicate with the department, so that arrangements can be made for the examination of the 'Neosalvarsan .9' in your possession.

"Dealers who fail to afford the department complete co-operation, and who are found to have this spurious product in their possession, will be summarily dealt with."—*New York Drug Bulletin.*

